# Hepatitis E virus infection in pigs: a first report from Zambia

**DOI:** 10.1080/22221751.2021.2002669

**Published:** 2021-11-21

**Authors:** Herman M. Chambaro, Michihito Sasaki, Walter Muleya, Masahiro Kajihara, Misheck Shawa, Kabemba E. Mwape, Hayato Harima, Yongjin Qiu, William W. Hall, Paul Fandamu, David Squarre, Edgar Simulundu, Hirofumi Sawa, Yasuko Orba

**Affiliations:** a Division of Molecular Pathobiology, International Institute for Zoonosis Control, Sapporo, Japan; b Virology Laboratory, Central Veterinary Research Institute, Lusaka, Zambia; c Department of Veterinary Services, Ministry of Fisheries and Livestock, Lusaka, Zambia; d Department of Biomedical Sciences, School of Veterinary Medicine, University of Zambia, Lusaka, Zambia; e Department of Clinical Studies, School of Veterinary Medicine, University of Zambia, Lusaka, Zambia; f National Virus Reference Laboratory, University College Dublin, Dublin, Ireland; g Global Virus Network, Baltimore, MD, USA; h Department of Conservation Science, Royal (Dick) School of Veterinary Studies, The University of Edinburgh, Edinburgh, UK; i Department of Disease Control, School of Veterinary Medicine, University of Zambia, Lusaka, Zambia; j Macha Research Trust, Choma, Zambia; k International Collaboration Unit, International Institute for Zoonosis Control, Hokkaido University, Sapporo, Japan; l One Health Research Center, Hokkaido University, Sapporo, Japan

**Keywords:** Hepatitis E virus, genotype 3, seroprevalence, domestic pig, Zambia

## Abstract

While evidence suggests presence of HEV infection in humans in Zambia, currently, there is no information on its occurrence in domestic pigs. Here, we investigated the presence of HEV antibodies and genome in domestic pigs in Zambia. Sera (*n* = 484) from domestic pigs were screened for antibodies against HEV by ELISA while genome detection in fecal (*n* = 25) and liver (*n* = 100) samples from slaughter pigs was conducted using nested RT–PCR assay. Overall, seroprevalence was 47.7% (231/484) while zoonotic genotype 3 HEV RNA was detected in 16.0% (20/125) of slaughtered pigs. This is the first report to highlight occurrence of HEV infection in domestic pigs in Zambia. This finding suggests possible contamination of the pork supply chain. Moreover, there is a potential risk of zoonotic transmission of HEV to abattoir workers, pig farmers and handlers.

Hepatitis E virus (HEV) causes hepatitis E, an emerging disease that is endemic in developing and developed countries worldwide. A number of animal species, including wild boar, pigs, camels, and deer are reservoirs for HEV [1–3]. Although HEVs belonging to the *Orthohepevirus A* species are known to infect humans, of the eight genotypes identified so far, only genotypes 3, 4 and 7 infect both humans and animals [2,4–8]. Genotype 3 (HEV-3) and 4 (HEV-4) HEV are predominant in pigs and wild boar populations while genotype 7 has been identified in camels [2,4]. However, HEV-3 and HEV-4 are largely responsible for sporadic cases of human hepatitis [2,7]. HEV is primarily transmitted through the fecal-oral route *via* contaminated food or water and is commonly associated with acute viral hepatitis in humans [2]. Occasional epidemics, mostly associated with poor sanitation and consumption of contaminated pork, have been reported in developing countries in Africa and Asia [2]. While the vast majority of infections remain asymptomatic, the risk of overt clinical hepatitis is high in immunocompromised patients and pregnant women [2]. In Zambia, HEV infection has been associated with human immunodeficiency virus (HIV) seropositivity [9], although its role in the high maternal mortality (183/100,000) is not yet understood. In this study, we report the first detection of HEV antibodies and genome in domestic pigs in Zambia.

A total of 484 serum samples were collected from adult domestic pigs in Lusaka and Eastern Province of Zambia between May 2017 and December 2019. Of these, 352 samples were obtained from Lusaka Province from crossbred pigs (landrace/large white) at Chibolya abattoir (*n* = 176) and exotic pigs (landrace) at a commercial farm (*n* = 176) while 132 samples were collected from indigenous free-range pigs in Katete District of Eastern Province. Additionally, fecal (*n* = 25) and liver (*n* = 100) samples were collected from pigs (*n* = 125) at Chibolya abattoir in December 2019.

Serologic analysis for HEV antibodies was performed with an Indirect Multi-species ELISA (ID-Vet, Grabels, France) which is based on the recombinant capsid protein of HEV-3. The ELISA has a reported test specificity of 100%. RNA was extracted from liver and fecal samples using the RNeasy Plus Mini Kit and fecal RNeasy PowerMicrobiome Kit (https://www.qiagen.com) as per manufacturer’s protocol. Screening for HEV genome was conducted by nested RT–PCR targeting the partial open reading frame (ORF) 1 and 2 genes [10,11]. Amplified products were purified and used for direct Sanger sequencing. Phylogenetic analysis was implemented in MEGA V7.0 (https://www.megasoftware.net). The sequences were deposited in GenBank under accession numbers LC653123-LC653139 (ORF1) and LC621322-LC621339 (ORF2). Simple logistic regression was used to model the dependency of HEV seropositivity on the pig management system (i.e. Confined, semi-confined and free-range).

Overall, seroprevalence was 47.7%, 95% CI [43.3–52.2], although this was significantly high (OR  =  2.0, 95% CI [1.3–3.1]) in slaughtered pigs from Chibolya abattoir (56.3%, 95% CI [48.9–63.4]) compared to that in pigs from a commercial farm (39.2%, 95% CI [32.2–46.6]). There was no significant difference (OR = 1.4, 95% CI [0.9–0.2]) in seroprevalence rates between indigenous free-range pigs from Katete District in Eastern Province (47.3%, 95% CI [39.4–56.2]) and crossbred pigs from Chibolya abattoir (56.3%, 95% CI [48.9–63.4]), possibly due to contrasting types of pig management systems. Pigs from Chibolya abattoir and Katete District are raised under semi-confined and free-range types of management systems, respectively. While the reasons for the observed high seroprevalence (47.7%) are not clear, the ELISA assay and type of pig management system may have contributed to the observed high seroprevalence. Similarly, as previously observed [5], seroprevalence rates are high in adult pigs compared to weaners. On logistic regression, only semi-confined pig management system was significantly associated with HEV seropositivity (OR = 2.0, 95% CI [1.3–3.1]; Supplementary Table 1).

HEV RNA was detected in both fecal (32%; 8/25) and liver (12%; 12/100) samples. However, the relatively low HEV RNA positivity (16%) in slaughtered pigs was probably due to virus clearance in adult pigs as previously observed [12]. Similarly, detection of viremic pigs is low, possibly due to transient viremia [12]. Phylogenetic analysis revealed that all sequences obtained in this study belonged to the zoonotic HEV-3 (Figure 1; Supplementary Figure 2). Topologically, in the partial ORF2 phylogeny, genotype 3 viruses from this study formed five distinct clusters (Figure 1). Furthermore, pairwise nucleotide identity matrix comparison showed high intragenotypic diversity within the ORF1 (91.9–99.6%; Supplementary Table 2) and ORF2 (91.9–99.7%; Supplementary Table 3) genes of HEVs detected in Zambia.

**Figure 1.**
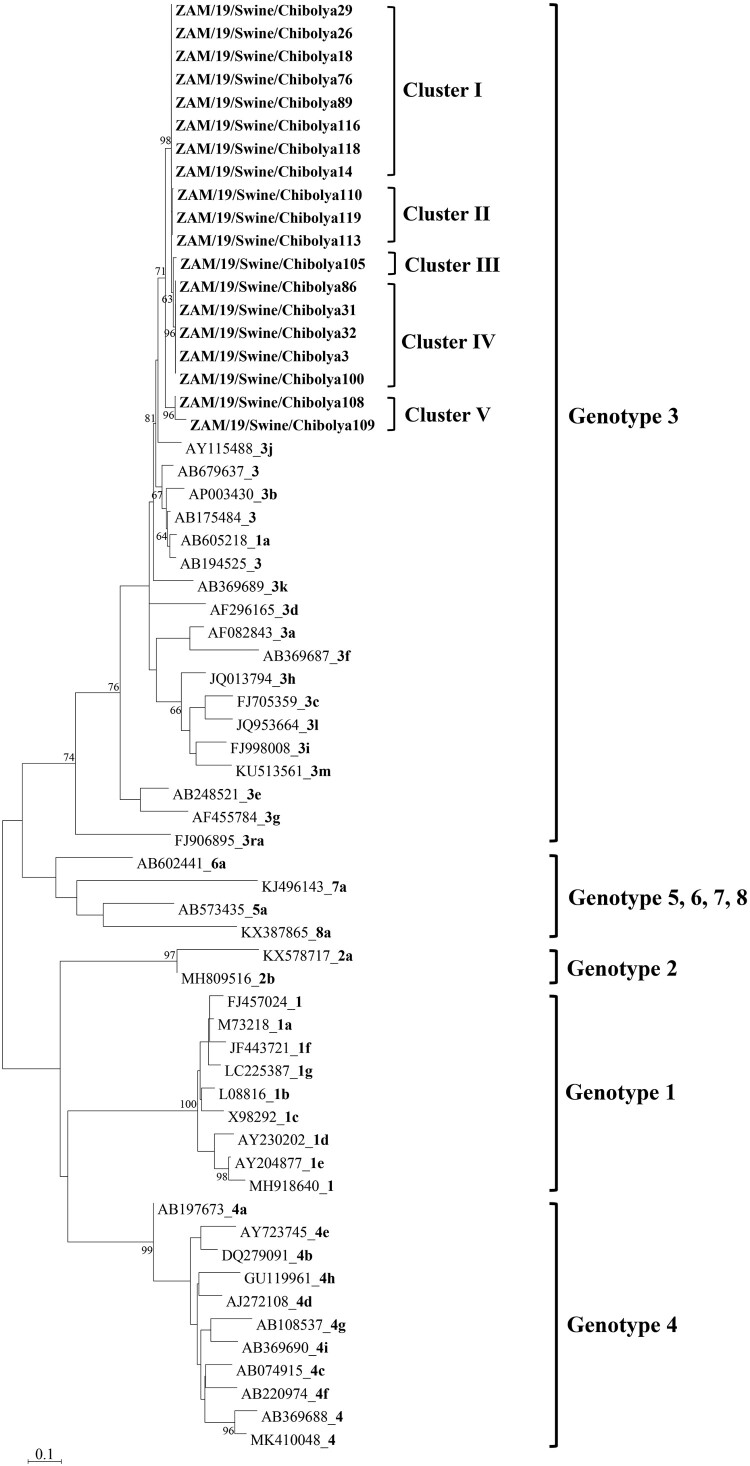
Phylogenetic tree of the partial (309 bp) ORF2 gene of hepatitis E viruses detected in domestic pigs in Zambia and reference sequences [15] retrieved from the GenBank (https://www.ncbi.nlm.nih.gov). The tree was generated in MEGA7 (https://www.megasoftware.net) using maximum likelihood method based on the Tamura-Nei model with 1000 bootstrap replicates. Bootstrap values >60% are indicated at the branch nodes. Viruses characterized in this study are indicated in bold text. Reference sequences are indicated by their accession number, followed by the genotype (number) and/or clade (letter) in bold text. Right brackets denote cluster or genotype. Bar, number of substitutions per site.

To our knowledge, this is the first report to highlight the occurrence of zoonotic HEV-3 infection in domestic pigs in Zambia. A similar study in Madagascar found probable evidence of virus transmission from infected pigs to abattoir workers [13]. Furthermore, HEV-3 was associated with viral hepatitis in patients in Egypt and Mayotte [8,14]. In Zambia, HEV infection was found to be a common occurrence in infants and adults, with a strong association to seropositive HIV status in adults [9]. The risk of HEV-3 infection, especially among abattoir workers and pig handlers in Zambia remains high. At unregulated abattoirs such as Chibolya abattoir in Lusaka Province, pigs are slaughtered and processed without any protective clothing and in poor sanitary conditions. Equally, zoonotic HEV-3 poses a public health risk to most poor, low-income households who largely depend on cheap pork from unregulated abattoirs. To further clarify the transmission dynamics of pig-associated HEV-3, more studies will need to be conducted in animals and humans, particularly among high-risk populations such as abattoir workers, pig farmers and handlers, immunocompromised patients and pregnant women in Zambia.

## Author contributions

Conceptualization, H.M.C., M.S., W.W.H., P.F., E.S., H.S., and Y.O., methodology, H.M.C., M.S., W.M., E.S., H.S., and Y.O., investigation, H.M.C., M.S., W.M., M.K., K.E.M., H.H., Y.Q., D.S., E.S., H.S., and Y.O., Data curation, H.M.C., M.S., W.M., M.K., M.S., E.S., H.S., and Y.O., Funding acquisition, H.M.C., M.S., W.W.H., P.F., E.S., H.S., and Y.O., supervision, M.S., W.W.H., E.S., H.S., and Y.O. All authors participated in revising and approving the final version of the manuscript.

## Supplementary Material

Supplementary_Table_2_clean_copy.docx

## Data Availability

The data that support the findings of this study are openly available in the NCBI GenBank at https://www.ncbi.nlm.nih.gov under accession numbers LC621322-LC621339.
