# Supplementary material for: Hepatitis E virus infection in pigs: a first report from Zambia
Source: Emerg Microbes Infect. 2021 Nov 21;10(1):2169–72. doi: 10.1080/22221751.2021.2002669 (PMC8635572; doi:10.1080/22221751.2021.2002669)
Supplement: Supplementary_Table_2_clean_copy.docx [file TEMI_A_2002669_SM8345.docx]

| ^†^**Sample Name** | 108 | 35 | 26 | 110 | 32 | 29 | 31 | 22 | 89 | 109 | 76 | 14 | 105 | 113 | 18 | 86 | 118 |
| --- | --- | --- | --- | --- | --- | --- | --- | --- | --- | --- | --- | --- | --- | --- | --- | --- | --- |
|  | **Pairwise identity (%)** | | | | | | | | | | | | | | | | |
| **108** |  | 93.4 | 93.4 | 93.4 | 93.4 | 93.4 | 93.4 | 93.4 | 93.4 | 93.4 | 93.4 | 93.4 | 93 | 93 | 93.7 | 93.7 | 91.9 |
| **35** | 93.4 |  | 100 | 100 | 100 | 100 | 100 | 100 | 100 | 100 | 100 | 100 | 99.6 | 99.6 | 98.9 | 97.4 | 97.8 |
| **26** | 93.4 | 100 |  | 100 | 100 | 100 | 100 | 100 | 100 | 100 | 100 | 100 | 99.6 | 99.6 | 98.9 | 97.4 | 97.8 |
| **110** | 93.4 | 100 | 100 |  | 100 | 100 | 100 | 100 | 100 | 100 | 100 | 100 | 99.6 | 99.6 | 98.9 | 97.4 | 97.8 |
| **32** | 93.4 | 100 | 100 | 100 |  | 100 | 100 | 100 | 100 | 100 | 100 | 100 | 99.6 | 99.6 | 98.9 | 97.4 | 97.8 |
| **29** | 93.4 | 100 | 100 | 100 | 100 |  | 100 | 100 | 100 | 100 | 100 | 100 | 99.6 | 99.6 | 98.9 | 97.4 | 97.8 |
| **31** | 93.4 | 100 | 100 | 100 | 100 | 100 |  | 100 | 100 | 100 | 100 | 100 | 99.6 | 99.6 | 98.9 | 97.4 | 97.8 |
| **22** | 93.4 | 100 | 100 | 100 | 100 | 100 | 100 |  | 100 | 100 | 100 | 100 | 99.6 | 99.6 | 98.9 | 97.4 | 97.8 |
| **89** | 93.4 | 100 | 100 | 100 | 100 | 100 | 100 | 100 |  | 100 | 100 | 100 | 99.6 | 99.6 | 98.9 | 97.4 | 97.8 |
| **109** | 93.4 | 100 | 100 | 100 | 100 | 100 | 100 | 100 | 100 |  | 100 | 100 | 99.6 | 99.6 | 98.9 | 97.4 | 97.8 |
| **76** | 93.4 | 100 | 100 | 100 | 100 | 100 | 100 | 100 | 100 | 100 |  | 100 | 99.6 | 99.6 | 98.9 | 97.4 | 97.8 |
| **14** | 93.4 | 100 | 100 | 100 | 100 | 100 | 100 | 100 | 100 | 100 | 100 |  | 99.6 | 99.6 | 98.9 | 97.4 | 97.8 |
| **105** | 93 | 99.6 | 99.6 | 99.6 | 99.6 | 99.6 | 99.6 | 99.6 | 99.6 | 99.6 | 99.6 | 99.6 |  | 99.3 | 99.3 | 97.8 | 98.2 |
| **113** | 93 | 99.6 | 99.6 | 99.6 | 99.6 | 99.6 | 99.6 | 99.6 | 99.6 | 99.6 | 99.6 | 99.6 | 99.3 |  | 98.5 | 97 | 97.4 |
| **18** | 93.7 | 98.9 | 98.9 | 98.9 | 98.9 | 98.9 | 98.9 | 98.9 | 98.9 | 98.9 | 98.9 | 98.9 | 99.3 | 98.5 |  | 98.5 | 97.4 |
| **86** | 93.7 | 97.4 | 97.4 | 97.4 | 97.4 | 97.4 | 97.4 | 97.4 | 97.4 | 97.4 | 97.4 | 97.4 | 97.8 | 97 | 98.5 |  | 98.2 |
| **118** | 91.9 | 97.8 | 97.8 | 97.8 | 97.8 | 97.8 | 97.8 | 97.8 | 97.8 | 97.8 | 97.8 | 97.8 | 98.2 | 97.4 | 97.4 | 98.2 |  |

**Supplementary Table 2.** Pairwise identity matrix based on nucleotide sequences of the partial (272 bp) ORF1 gene of HEVs detected in Zambia

^†^ZAM/19/SWINE/CHIBOLYA; %, Percent
